# The Cervical Microbiome in Hispanic Populations in Texas and Puerto Rico with and without Cervical Dysplasia

**DOI:** 10.21203/rs.3.rs-8002153/v1

**Published:** 2026-06-10

**Authors:** D’Shaunique Walters, Molly B. El Alam, Timothy Harris, David Lo, Jhoan Sebastian Gonzalez Diaz, Tatiana Cisneros Napravnik, Erica J. Lynn, Laurel D. Boatright, Dalissa Negron Figueroa, Allison Judge, Christy Charles John Bhaskar, Antoine Fontillas, Shafqat Fahmid Ehsan, Rui Wang, Shae N. Jansen Aref, Nadim J. Ajami, Jagannadha Sastry, Travis T. Sims, Josefina Romaguera, Stephanie Dorta-Estremera, Ann Klopp, Filipa Godoy-Vitorino, Lauren E. Colbert

**Affiliations:** The University of Texas MD Anderson Cancer Center; The University of Texas MD Anderson Cancer Center; The University of Texas MD Anderson Cancer Center; The University of Texas MD Anderson Cancer Center; The University of Texas MD Anderson Cancer Center; The University of Texas MD Anderson Cancer Center; The University of Texas MD Anderson Cancer Center; The University of Texas MD Anderson Cancer Center; The University of Texas MD Anderson Cancer Center; The University of Texas MD Anderson Cancer Center; The University of Texas MD Anderson Cancer Center; The University of Texas MD Anderson Cancer Center; The University of Texas MD Anderson Cancer Center; The University of Texas MD Anderson Cancer Center; The University of Texas MD Anderson Cancer Center; The University of Texas MD Anderson Cancer Center; The University of Texas MD Anderson Cancer Center; The University of Texas MD Anderson Cancer Center; University of Puerto Rico, Medical Sciences Campus; University of Puerto Rico School of Medicine, Medical Sciences Campus; The University of Texas MD Anderson Cancer Center; University of Puerto Rico School of Medicine, Medical Sciences Campus; The University of Texas MD Anderson Cancer Center

**Keywords:** cervical microbiota, cervical dysplasia, human papillomavirus (HPV), 16S rRNA sequencing

## Abstract

Within the United States, Hispanic women, especially those in Puerto Rico, face an increased risk of cervical cancer development. The objective of this study was to explore the cervical microbiota of Hispanic women at high risk of developing HPV-induced cervical dysplasia or with cervical dysplasia treated in Texas and Puerto Rico. Cervical swab samples were collected from 296 participants (N = 80 Texan Non-Hispanic White, N = 98 Texan Hispanic, and N = 118 Puerto Rican Hispanic) during each patient’s initial visit and subjected to 16S V4 rRNA gene sequencing for microbiome profiling. HPV types were grouped as HPV 16, other high-risk HPV types, and other using HPV genotyping. Among participants, 71% (N = 211) were classified as high-risk normal, and 29% (N = 85) had cervical dysplasia. HPV 16 was detected in 15% (N = 45), other high-risk HPV types in 33% (N = 98), while 52% (N = 152) of patients were classified as “other”. Comparative analysis of microbial community structures across locations revealed distinct compositions, with Texan Hispanic women showing higher alpha diversity for two alpha diversity metrics (Pielou evenness and Shannon Diversity Index). The prevalence of CSTs varied across locations and disease states, with CSTs III and IV-B being among the most common in the study cohorts. Overall, this descriptive study provides a better understanding of cervical microbiome in Hispanic women across multiple geographic locations, in order to guide future interventions.

## Introduction

Cervical cancer is the fourth most common cancer diagnosed among women worldwide. It accounts for approximately 660,000 cancer-related diagnoses in 2022 and is the fourth leading cause of cancer-related deaths among women worldwide.^1,2^ The incidence of cervical cancer from 2001 to 2017 was shown to have increased from 9.2 to 13.0 per 100,000 person-years.^3^ Within the United States (U.S.), over 33.7 million individuals reside in Texas and Puerto Rico combined. The Hispanic or Latino population accounts for 50% or higher in some regions of Texas and comprises over 95% of the estimated 3 million individuals residing in Puerto Rico.^4,5^ Among Hispanic Americans, cancer is a leading cause of death, with women having a higher incidence rate for cervical cancer development compared to non-Hispanic White women. Hispanic women in Puerto Rico face a notably higher incidence of cervical cancer, exceeding rates observed in other U.S. regions.^6^ The development of cervical cancer is a long process beginning with its precursor, cervical dysplasia, progressing through stages from mild to severe intraepithelial neoplasia and driven by various factors.

The development of cervical cancer is influenced by different factors related to both the host and microbiota.^7,8^ The vaginal and cervical microbiomes are complex ecosystems of microorganisms, encompassing bacteria, fungi, and viruses, with variations in bacterial populations observed between sites.^9,10^ *Lactobacilli* species, particularly *Lactobacillus crispatus*, usually dominate these ecosystems, forming a regulated community often considered a “healthy” microbial environment. Recent studies suggest that the vaginal microbiome of women of European ancestry exhibits lower microbial diversity, with a higher abundance of *Lactobacilli crispatus* compared to African American women, suggesting that race may influence the vaginal ecosystem.^11^ With the development of next-generation sequencing approaches, such as 16S rRNA gene sequencing, the vaginal microbiome has been classified into one of five community state types (CSTs), depending on its composition and the relative abundance of different bacterial species. CST-I, CST-II, CST-III, and CST-V are dominated by the abundance of various *Lactobacillus* species, including *L. crispatus*, *L. gasseri*, *L. iners*, and *L. jensenii*, respectively. CST-IV represents a diverse microbiota composed of various anaerobic bacterial species and can be further sub-classified depending on the most abundant taxa present.^12,13^ Similarly, these CST classifications have been used to characterize the cervical microbiome. Despite the crucial role in protecting against foreign organisms, such as through the production of lactic acid, not all *Lactobacilli* species in this genus are associated with “healthy” microbial ecosystems or normal cytology.^14^ For example, a meta-analysis found CSTs dominated by *L. iners* or non-*Lactobacilli* species were associated with a higher prevalence of cervical dysplasia and human papillomavirus (HPV), the primary etiological agent of cervical cancer.^15^

Within the United States, among women aged 15 to 59, the incidence of any HPV infection was 1,227 per 10,000, estimating roughly 11.8 million individuals in 2018. Notably, 636 per 10,000 women experienced a disease-associated HPV infection.^16^ With over 200 genotypes classified, some genotypes are strongly linked to cancer; these 15 genotypes are known as “high-risk HPVs,” with HPV16 as the predominant genotype linked to cervical cancer.^17–19^ HPV-mediated cervical cancers develop in a stepwise manner, with persistent HPV infection leading to the development of intraepithelial lesions, and further progression to cervical cancer driven by the activity of viral oncoproteins, which disrupt normal cell cycle processes, inhibit apoptosis, and lead to genetic instability.^20,21^ Presently, there are only three HPV vaccines approved by the FDA: Cervarix, Gardasil, and Gardasil 9. The efficacy of these vaccines tethers on the HPV genotype being included in the vaccine-preventable genotypes.^22^ Taken together, this evidence suggests a strong link between patient ethnicity, the cervical microbiome, HPV infection, and cervical dysplasia. However, it is unclear how geographic locations influence the cervical microbiota among women of the same ethnic background. This study aims to explore the cervical microbiome profiles of Hispanic women in Texas and Puerto Rico, who are at risk for HPV-induced cervical dysplasia or clinically diagnosed with cervical dysplasia, using next-generation sequencing of the 16S rRNA gene region V4, based on cervical swabs.

## Results

### Patient demographics and clinical characteristics

The study included 296 patients with and without cervical dysplasia (Table 1). Of these patients, 60.1% (N = 178) were residing in Texas, and 39.9% (N = 118) were treated in Puerto Rico. 71.3% (N = 211) were categorized as high-risk normal, of whom 25.6% (N = 54) identified as Texan Non-Hispanic White, 32.2% (N = 68) identified as Texan Hispanic, and 42.2% (N = 89) identified as Puerto Rican Hispanic. Eighty-five patients (28.7%) were clinically diagnosed with cervical dysplasia, of whom 30.6% (N = 26) identified as Texan Non-Hispanic White, 35.3% (N = 30) identified as Texan Hispanic, and 34.1% (N = 29) identified as Puerto Rican Hispanic. HPV 16 was detected in 45 patients (15.2%), and other high-risk HPV genotypes were detected in 98 patients (33.1%). Among patients who tested positive for HPV, 83 (52.9%) had only one HPV genotype detected, while 74 (47.1%) exhibited multiple genotypes (**Supplemental Table 1**). In a stratified analysis for each disease state, we observed that among high-risk normal women, multiple HPV genotypes were detected in 13.7% (N = 7) of Texan Non-Hispanic White women, 29.4% (N = 15) of Texan Hispanic women, and 56.9% (N = 29) of Puerto Rican Hispanic women. For women with cervical dysplasia, multiple HPV genotypes were detected in 30.4% (N = 7) of Texan Non-Hispanic White women, 30.4% (N = 7) of Texan Hispanic women, and 39.1% (N = 9) of Puerto Rican women. Fifty-six patients (35.7%) possessed genotypes covered by the available HPV vaccines, whereas 101 (64.3%) exhibited genotypes not encompassed by the vaccine.

### Geographic differences in cervical microbial profiles

**Figure 1:**
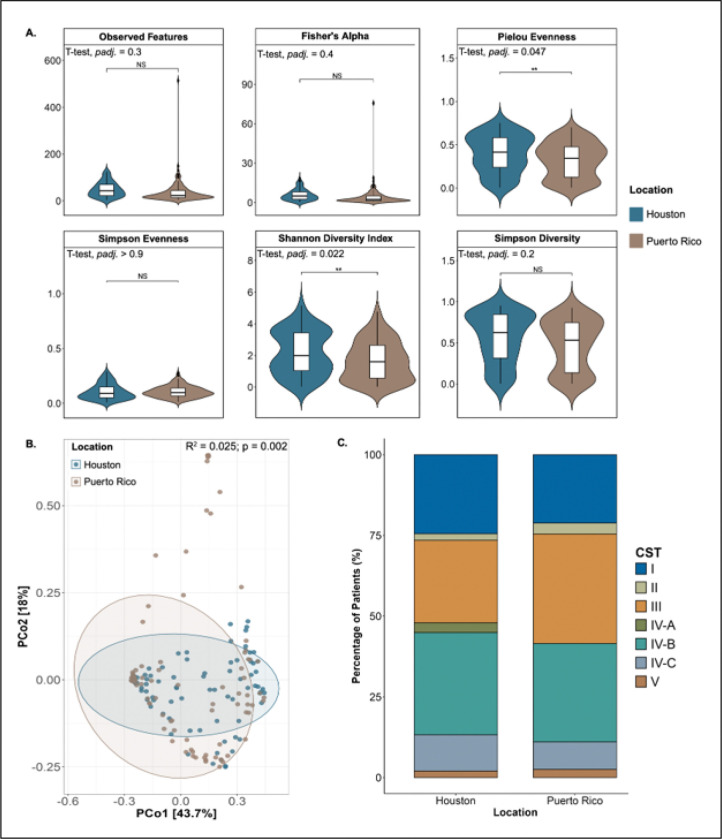
Cervical microbiome community composition association with patient geographic location for Hispanic individuals. (A) Alpha diversity violin boxplot (Observed Features, Fisher’s Index, Pielou’s Evenness, Simpson’s Evenness, Shannon Diversity Index, and Simpson Diversity). (B) Principal coordinate analysis of beta diversity employing weighted UniFrac, along with subsequent analysis using PerMANOVA. (C) Stacked bar plot summarizing the distribution of cervical community states (CSTs) across both geographic locations. *Abbreviations*: CST = community state types.

To examine the variations within the microbial community composition across geographic locations, we analyzed species diversity within the cervical microbiome among Hispanic women. We observed significantly higher alpha diversity in the Texan patients as compared to the Puerto Rico patients, specifically in the Pielou evenness and Shannon Diversity indices (Figure 1A). The evaluation of beta diversity revealed a significant difference in the composition between the two locations (Figure 1B; weighted UniFrac, R^2^ = 0.025, *p* = 0.002). The three most prevalent CSTs among both the Texan and Puerto Rican groups were I, III, and IV-B (Figure 1C). Specifically, in Texan patients, the most prevalent CST was IV-B (31.6%), while in the Puerto Rican group, the top CST was III (33.9%).

### Microbial community structure and disease state

**Figure 2:**
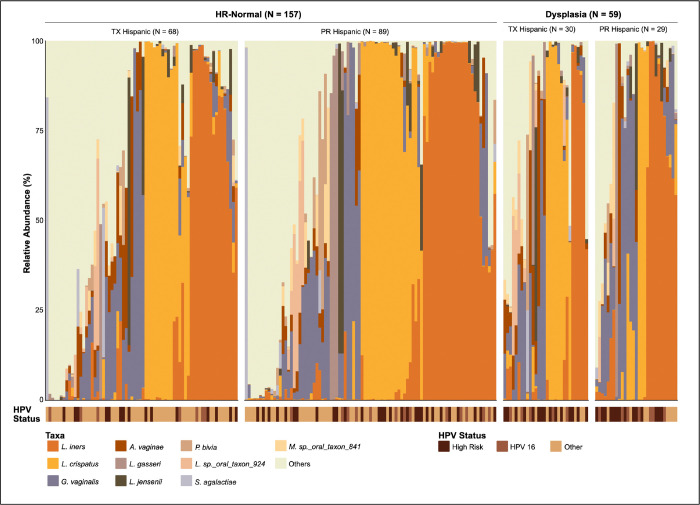
Species-level classification of microbiome profiles of high-risk normal and cervical dysplasia individuals. *Abbreviations*: TX = Texas; PR = Puerto Rico; HR-Normal = high-risk normal; HPV = human papillomavirus.

The three most abundant bacterial species identified across both disease states were *Lactobacillus iners*, *Lactobacillus crispatus*, and *Gardnerella vaginalis* (Figure 2). To determine whether the disease state affects the composition of the cervical microbiome community, we assessed the microbial structure using PerMANOVA and found no significant difference between the two groups (weighted UniFrac, R^2^ = 0.005, *p* = 0.32)(**Supplemental Figure 1A)**. The top prevalent CSTs within the high-risk normal participants were III (30.6%), IV-B (28.0%), and I (22.9%), versus IV-B (39.0%), III (28.8%), and I (22.0%) in the cervical dysplasia group (**Supplemental Figure 1B**).

**Figure 3:**
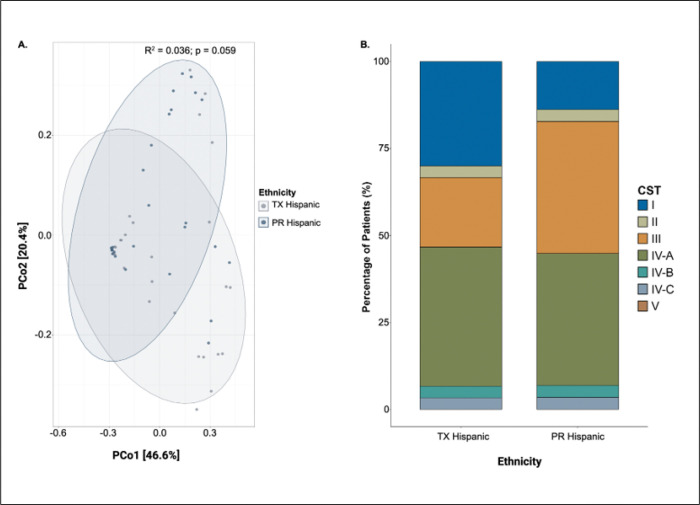
Cervical microbiome community structure and CST distribution for Hispanic dysplasia individuals. (A) Principal coordinate analysis of beta diversity employing weighted UniFrac by ethnicity along with subsequent analysis using PerMANOVA. (B) Stacked bar plot summarizing the distribution of CSTs across the two ethnic groups in dysplasia participants. *Abbreviations*: TX = Texas; PR = Puerto Rico; CST = community state types.

The most abundant bacterial species identified within the high-risk normal participants were *Lactobacillus iners* (N = 147 patients, mean = 0.25 (SD 0.38)), *Lactobacillus crispatus* (N = 135 patients, mean = 0.21 (SD 0.37)), and *Gardnerella vaginalis* (N = 125 patients, mean = 0.11 (SD 0.21)). The three most abundant bacterial species identified within the cervical dysplasia participants were *Lactobacillus iners* (N = 52 patients, mean = 0.26 (SD 0.37)), *Lactobacillus crispatus* (N = 50 patients, mean = 0.20 (SD 0.35)), and *Gardnerella vaginalis* (N = 53 patients, mean = 0.17 (SD 0.22)). Next, we evaluated whether ethnicity influences the cervical microbiome composition of patients with dysplasia. We observed no significant differences in alpha diversity metrics across the three ethnic groups (*padj.* > 0.05) (Table 2). To study the differences in the microbial composition of patients with cervical dysplasia, we performed a subset analysis included Hispanic patients with cervical dysplasia (**Supplemental Table 2**). We found no difference in the community structure of the cervical microbiome between the two ethnic groups (weighted UniFrac, R^2^ = 0.036, *p* = 0.06) (Figure 3A). Among women with cervical dysplasia, the most prevalent CSTs were IV-B (40%) among Texan Hispanic women, and both III and IV-B (37.9%) among Puerto Rican Hispanic women (Figure 3B).

### Associations between HPV status and microbial profiles

**Figure 4:**
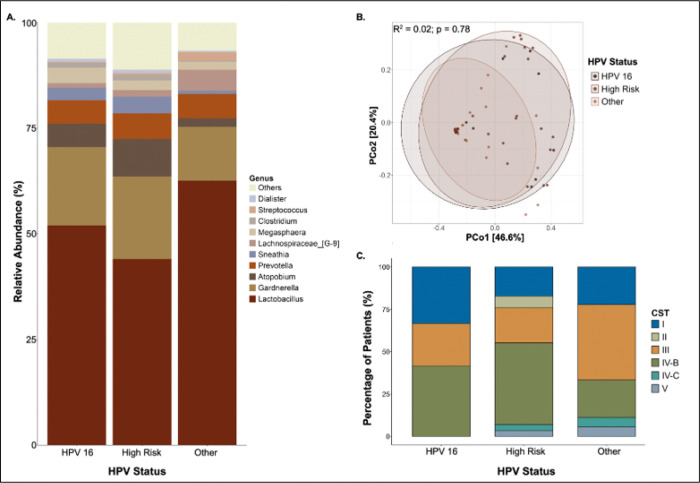
Cervical microbiome community composition association with HPV infection in Hispanic women. (A) Microbiome composition on genus-level by HPV status. (B) Principal coordinate analysis of beta diversity employing weighted UniFrac, along with subsequent analysis using PerMANOVA. (C) Stacked bar plot summarizing the distribution of cervical community states (CSTs) across HPV groups. *Abbreviations*: CST = community state types.

The two most abundant bacterial genera identified across HPV states were *Lactobacillus* and *Gardnerella* (Figure 4A). To examine the variations within the microbial community composition, we analyzed species diversity within the cervical microbiome communities across the three HPV groups among Hispanic women with dysplasia. We observed no significant differences in alpha diversity metrics across the three HPV groups (Table 3). On principal coordinate analysis, we found no differences in the microbiome composition of patients with cervical dysplasia across HPV groups (weighted UniFrac, R^2^ = 0.02, *p* = 0.78) (Figure 4B). The three most prevalent CSTs among the three HPV groups were I, III, and IV-B (Figure 4C). Specifically, in HPV 16-positive patients, the distribution was IV-B (41.7%), I (33.3%), and III (25.0%), while in patients with other high-risk HPV genotypes detected the top three were IV-B (48.3%), III (20.7%), and I (17.2%). We observed III (44.4%) as the most common CST, followed by I and IV-B (22.2%), in patients with HPV low-risk genotypes, HPV-negative, and unknown HPV status.

## Discussion

This study explores the cervical microbiome profile among women of the same ethnic background, specifically Hispanic women, who exhibit an increased susceptibility to cervical cancer development compared to white Americans, from different geographic locations. Our findings demonstrate geographic differences in the cervical microbiome profiles of Hispanic women, highlighted by significant differences in composition between Texas and Puerto Rico. Notably, we found no significant difference in the microbial community structure between disease states. Our data also demonstrate that *Lactobacilli* species, including *L. crispatus* and *L. iners*, dominate the cervical microbiome within the study cohort, with most individuals belonging to a CST III or IV-B profile. Taken together, our findings illustrate spatial variations in the cervical microbiota of Hispanic women and emphasize the importance of understanding these differences to further investigate disease progression toward cervical cancer.

The cervicovaginal microbiota is generally dominated by *Lactobacillus* species, including the most common species: *L. crispatus*, *L. gasseri*, *L. iners*, and *L. jensenii*.^12^ In alignment with this knowledge, *Lactobacilli* species, including *L. crispatus* and *L. iners*, dominate the cervical microbiome within the study cohort. The presence of Lactobacillus species plays a critical role in maintaining the balance of the cervicovaginal ecosystem through the production of antibacterial and antiviral agents, including lactic acid, bacteriocins, and hydrogen peroxide, which leads to a reduction of pH, thereby inhibiting the growth of pathogenic bacteria and viruses.^23^ Previous studies have demonstrated that alterations in the cervicovaginal microbial flora are strongly connected with numerous gynecologic conditions, including cervical cancer. For example, the progression of cervical dysplasia toward cervical cancer is associated with a shift in the microbiome composition, highlighted by an increase in alpha diversity and a reduction of *Lactobacillus*.^24^

The structure of this dynamic ecosystem is shaped by race and ethnicity, as studies have observed variations in microbial prevalence across ethnic groups, with some microbial profiles being more prevalent within certain groups.^11,12,25^ Lactobacillus-dominant microbial profiles are more common among Asian and White women compared to Black and Hispanic women. Notably, Hispanic women exhibit a CST-IV profile, characterized by a diverse microbiota with various anaerobic bacterial species.^12^ Using a Chinese population, Yang et al. demonstrated differences in CST distribution across different geographic locations, whereas *L. iners*-dominant profiles were prevalent in the Southern region compared to the Northern regions, which exhibited *L. crispatus*-dominant profiles at higher prevalence.^26^ This study identified that most Texan Hispanic women exhibited a CST IV profile, particularly sub-group CST IV-B, characterized by a high abundance of the genus *Gardnerella,* followed by CST III. On the other hand, most Puerto Rican Hispanic women were classified into CST III, followed by CST IV-B. Specifically, for Hispanic women residing in Puerto Rico, CST IV is more common across women with normal cytology and cervical dysplasia, which is associated with an increase in pro-inflammatory cytokines. Similarly, one study of 396 women from four main ethnic groups revealed that CSTs IV and III are more prevalent among Hispanic women than white women.^12^ In a smaller cohort study of Puerto Rican women, Tosado-Rodríguez et al. showed that the microbial profile of Puerto Rican Hispanic women was dominated by CST IV-C.^27^ However, these variances may be attributed to the larger sample size utilized in our study. The disparities observed in the sub-optimal profiles among these groups of Hispanic women can also be attributed to other lifestyle activities that are beyond the scope of this study; however, this could be considered a limitation.

Beyond race and ethnicity, various factors, including menstruation, influence the composition of the cervical microbiome.^28,29^ For instance, an elevation in *non-Lactobacillus*, specifically facultative anaerobes, such as *Gardnerella vaginalis*, has been linked to HPV infection in individuals with normal cytology.^30^ These findings support the hypothesis that changes in the cervicovaginal microbiota reduce Lactobacilli species, which, in turn, increase the risk of acquiring HPV and, consequently, developing cervical cancer. Within the United States, approximately 6.1 million women experienced a disease-associated HPV infection in 2018 despite the availability of three FDA-approved HPV vaccines.^16^ Although these three vaccines are used globally, only Gardasil 9 provides protection against nine out of the fifteen high-risk genotypes.^22^ However, multiple studies have indicated variability in the prevalence of HPV genotypes, contingent upon patient demographics such as geographic location, race, and ethnicity.^18,31–35^ In Puerto Rico, numerous studies have shown that genotypes not covered by existing vaccines are present among women.^34,36,37^ This is in line with our findings, where we observed the presence of genotypes not covered by the available HPV vaccines in more than half of patients with cervical dysplasia. These results highlight the limited efficacy of the currently available HPV vaccines as they lack coverage of HPV genotypes that are prevalent in these populations.

In summary, our findings illustrate spatial variations in the cervical microbiota of Hispanic women, who have been shown to have an increased susceptibility to cervical cancer development compared to white Americans. Cervical cancer and its precursor, cervical dysplasia, are impacted by the cervical microbiota, which, in turn, is modulated by the ethnic background of the patient. The presented study reveals the predominant presence of *Lactobacilli* species in the complex cervical microbial milieu of Hispanic women in Texas and Puerto Rico, with CSTs IV and III being the top two vaginal community state types, consistent with previous research findings. Our findings also suggest the need for broader coverage HPV vaccines, as high-risk HPV genotypes not covered in the available immunization are prevalent in Hispanic women, thereby contributing to the development of cervical dysplasia. While dietary habits, menstrual patterns, and sexual activity were not directly assessed in this study, these factors could potentially impact the cervical microbiome. Future research should incorporate these variables to understand their influence on microbial ecosystems and disease outcomes. Such studies will be critical in refining cervical cancer prevention strategies and improving the outcomes for Hispanic women.

## Methods

### Participant Recruitment and Sample Collection

#### The University of Texas MD Anderson Cancer Center and Harris Health System

Participants were enrolled at The University of Texas MD Anderson Cancer Center or Harris Health System, Lyndon B. Johnson General Hospital Oncology Clinic (N = 178) under Institutional Review Board (IRB)-approved protocol MDACC 2019–1059. Written informed consent was obtained from all participants in accordance with approved protocol guidelines. Inclusion criteria included participants being at least 18 years of age, diagnosed as having pre-malignant cervical lesions (i.e., receiving colposcopy, Loop Electrosurgical Excision Procedure (LEEP), cone, or pap test for evaluation of dysplasia and/or HPV positivity), or a diagnosis in the past year of cervical intraepithelial neoplasia.

Cervical swab samples were collected during a gynecological exam using Isohelix Buccal Swabs (Isohelix, cat. #SK-3S). Physicians swabbed the cervix in a rotational motion, then submerged the swab into a BuccalFix Tube (Isohelix, cat. #BFX/S1–05-50) before transport. Samples were transported at room temperature to the lab within 4 hrs of collection, then vortexed for 15 s and stored at −80°C until analysis.

#### University of Puerto Rico and San Juan City Hospital

Study participants (N = 118) were enrolled at the University of Puerto Rico and San Juan City Hospital under IRB protocol #1050114. All participants were HIV negative, and exclusion criteria included active urinary infections, history of common urinary incontinence, sexually transmitted diseases (STDs), antibiotics taken in the prior month, candidiasis, being less than 21 or more than 60 years old, and treatment for or suspicion of prior toxic shock syndrome. Written informed consent was obtained from all participants, and recruitment was conducted in compliance with the regulations of the Human Subjects Protection Office at the University of Puerto Rico and *the Declaration of Helsinki.*

Cervical swab samples were collected using Sterile Catch-All Specimen Collection Swabs (Epicentre Biotechnologies, Madison, WI, United States). The physician inserted the swab into the cervix, rotated it along the lumen in a circular motion, and then placed it in capped, sterile 2 mL microtubes. Swabs were kept for 1–4 hrs in a cooler with ice packs before transportation to the lab, then stored at −80°C before analysis.

All patient demographic and clinical characteristics were obtained from electronic medical records and are summarized in Table 1. Patients were categorized as having dysplasia or high-risk normal. All individuals in the high-risk normal group were identified to be at high risk for developing HPV-induced cervical cancer due to a history of HPV infection or abnormal colposcopy, LEEP, cone, or pap smear.

### DNA Extraction

#### The University of Texas MD Anderson Cancer Center

DNA was isolated by the Alkek Center for Metagenomics and Microbiome Research at Baylor College of Medicine using the MagAttract PowerSoil DNA kit (#27100; QIAGEN, Germantown, MD) following the manufacturer's instructions.

#### University of Puerto Rico

DNA was extracted from swabs using the MoBIO PowerSoil DNA Isolation Kit (Qiagen, cat. #12888) per the manufacturer's instructions with the following modifications. The powerhead tubes were homogenized horizontally for 2 min at 2,000 rpm using a PowerLyzer^™^ 24 Bench Top Bead-Based Homogenizer (MoBio). During the lysis step, 100 μl of solution C2 and 100 μL of solution C3 were combined and vortexed for 5 s.

### HPV Genotyping

#### The University of Texas MD Anderson Cancer Center

For each patient, HPV status was extracted from medical records only if the results were within 3 months of swab collection. The presence of HPV 16 took priority over other high-risk and low-risk types in patient categorization. Patients with other high-risk types (HPV 18, 26, 31, 33, 35, 39, 45, 51, 52, 56, 58, 59, 68, 69, 73, and 82) were categorized as high-risk regardless of the presence of low-risk types. The absence of all 17 high-risk types or unknown status was categorized as other. In cases where HPV genotyping was unavailable or outside the range of 3 months, cervical swabs underwent genotyping using one of the following methods.

##### Atila Biotechnologies Kit

Swab DNA was assayed for 15 high-risk HPV genotypes via the AmpFire Multiplex HPV assay (#GHPV-100; ATILA BioSystems, Mountain View, CA). This isothermal qPCR method detects the following HPV types: 16, 18, 31, 33, 35, 39, 45, 51, 52, 53, 56, 58, 59, 66, and 68. Manufacturer instructions for isolated DNA were followed, and the Applied Biosystems 7500 Fast System SDS, version 1.5.1, software program was used to analyze the results.

##### VirMAP

The approach, fully described in Sammouri et al., was used to identify HPV genotypes in samples using shotgun metagenome sequencing and analyzed using VirMAP.^38^

#### University of Puerto Rico

The Labo Biomedical short polymerase chain reaction fragment assay (Labo Biomedical Products, Rijswijk, Netherlands, licensed Innogenetics technology) was used for HPV genotyping in swab DNA, as previously described in van Alewijk, D. et al. and Vargas-Robles, D. et al.^37,39^ This assay allows for the identification of the following common mucosal HPV genotypes: 14 high-risk types (HPV 16, 18, 31, 33, 35, 39, 45, 51, 52, 56, 58, 59, 66, and 68/73) and 11 low-risk types (HPV 6, 11, 34, 40, 42, 43, 44, 53, 54, 70, and 74).

### Sequencing and Analysis of 16S rRNA genes

16S rRNA genes were sequenced using methods adapted from the Human Microbiome Project.^40^ The 16S rRNA V4 hypervariable region was amplified via PCR using a 515F-806R primer pair and sequenced on the MiSeq platform using a 2 × 250 bp paired-end protocol. QIIME2, a microbiome bioinformatics platform, was used to process the 16S rRNA gene reads (v2023.7).^41^ The Earth Microbiome Project (EMP) protocol was employed to demultiplex the sequencing data.^42^ Sequence quality control was conducted employing DADA2 denoising. The trim and truncation parameters were selected based on quality score plots generated in QIIME2. Subsequently, an Amplicon Sequence Variant (ASV) feature table was assembled. A custom taxonomic classifier, trained on a cervicovaginal-specific database, was utilized for species-level classification.^43^ Taxonomy was assigned using a custom cervicovaginal classifier ^43^

The characterization of variations in species diversity within the microbial ecosystem was conducted using six distinct alpha diversity metrics computed with QIIME2. These metrics comprise Observed Features, Fisher’s Alpha, Pielou’s Evenness, Simpson’s Evenness, Shannon Diversity Index, and Simpson Diversity. Each sample was classified into community state types (CST) based on the composition of the cervical microbial community using the VAginaL community state typE Nearest CentroId clAssifier (VALENCIA).^44^ The characterization of differences between microbial ecosystems, beta diversity, was assessed using weighted UniFrac, which reflects the differences between microbial communities by considering the phylogenetic relationships and abundance of taxa, with distances calculated using the R package, microeco.^45,46^ All analyses presented in this study were conducted on unrarefied data.

### Statistical Analysis

The differences in the six alpha diversity indices between the two collection sites, representing Texan and Puerto Rican Hispanics, were examined using the t-test and adjusted for multiple comparisons using Bonferroni correction. The differences in alpha diversity indices among the three ethnic groups for patients clinically diagnosed with cervical dysplasia were evaluated using a Kruskal-Wallis rank-sum test and subsequently corrected employing Bonferroni correction. Comparison of the six alpha diversity indices between the three HPV groups of Hispanic patients with dysplasia was evaluated using a Kruskal-Wallis rank-sum test and subsequently corrected employing Bonferroni correction. Principal coordinate analysis (PCoA) and permutational multivariate analysis (PerMANOVA) were employed to compare beta diversity, utilizing the microeco package in R. All statistical analyses were conducted using R (version 4.4.2; RStudio 2025.09.1+401) and visualization was prepared using BioRender (https://BioRender.com). p-value < 0.05 was considered statistically significant for all analyses.

## Supplementary Material

This is a list of supplementary files associated with this preprint. Click to download.

• U54ManuscriptSupplementaryInformation.docx

## Figures and Tables

**Table 1.** Clinical status and patient characteristics of the study cohort.

	TX Non-Hispanic White	TX Hispanic	PR Hispanic
	(N = 80)	(N = 98)	(N = 118)
**Age** *(yrs.)*			
Mean (SD)	54.6 (13.3)	44.9 (10.8)	40.2 (10.9)
**BMI** *(kg/m^2^)*			
Mean (SD)	28.4 (6.1)	31.6 (8.9)	29.5 (7.2)
**Smoking Status**			
No	49 (61.3)	77 (78.6)	104 (88.1)
Yes	25 (31.3)	18 (18.4)	11 (9.3)
Unknown	6 (7.5)	3 (3.1)	3 (2.5)
**Disease Status**			
HR-Normal	54 (67.5)	68 (69.4)	89 (75.4)
Dysplasia	26 (32.5)	30 (30.6)	29 (24.6)
**Histology** *			
LGSIL	15 (57.7)	22 (77.3)	12 (41.4)
HGSIL	11 (42.3)	8 (26.7)	17 (58.6)
**HPV Status**			
HPV 16	13 (16.3)	12 (12.2)	20 (16.9)
High-Risk ^†^	25 (31.3)	30 (30.6)	43 (36.4)
Other ^‡^	42 (52.2)	56 (57.1)	55 (46.6)

*Abbreviations*: TX = Texas; PR = Puerto Rico; SD = standard deviation; BMI = body mass index; HR-Normal = high-risk normal; LGSIL = low-grade squamous intraepithelial lesion; HGSIL = high-grade squamous intraepithelial lesion; HPV = human papillomavirus

* Histology for participants clinically diagnosed with cervical dysplasia

† High-risk includes HPV 18, 26, 31, 33, 35, 39, 45, 51, 52, 56, 58, 59, 68, 69, 73, and 82

‡ Other includes HPV low-risk genotypes, HPV-negative, and unknown HPV status

**Table 2.** Alpha diversity metrics of cervical microbiome based on ethnicity in patients with cervical dysplasia. The Kruskal-Wallis rank-sum test was used to compare alpha diversity across the three ethnic groups for dysplasia participants. Values in boldface indicate FDR Adj. P < 0.05.

	TX Non-Hispanic White (N = 26)	TX Hispanic (N = 30)	PR Hispanic (N = 29)	P value	FDR Adj. P value^‡^
**Observed Features**				0.024	0.15
Mean (SD)	40.8 (31.7)	50.1 (30.3)	32.0 (25.4)		
**Fisher’s Alpha**				0.019	0.11
Mean (SD)	5.1 (4.3)	6.0 (4.1)	3.6 (3.2)		
**Pielou Evenness**				0.6	> 0.9
Mean (SD)	0.4 (0.2)	0.4 (0.2)	0.3 (0.2)		
**Simpson Evenness**				0.6	> 0.9
Mean (SD)	0.1 (0.1)	0.1 (0.1)	0.1 (0.1)		
**Shannon Diversity Index**				0.4	> 0.9
Mean (SD)	1.9 (1.3)	2.2 (1.4)	1.7 (1.0)		
**Simpson Diversity**				0.6	> 0.9
Mean (SD)	0.5 (0.3)	0.6 (0.3)	0.5 (0.3)		

*Abbreviations*: TX = Texas; PR = Puerto Rico; SD = standard deviation

‡ FDR Adj. P-value indicates a comparison of Texan Non-Hispanic White versus Texan Hispanic versus Puerto Rican.

**Table 3.** Alpha diversity metrics of cervical microbiome based on HPV status in Hispanic patients with cervical dysplasia. The Kruskal-Wallis rank-sum test was used to compare alpha diversity across the three ethnic groups for dysplasia participants. Values in boldface indicate FDR Adj. P < 0.05.

	HPV 16 (N = 12)	High-Risk (N = 29)	Other (N = 18)	P value	FDR Adj. P value^‡^
**Observed Features**				0.8	> 0.9
Mean (SD)	39.2 (32.0)	40.2 (29.3)	44.2 (28.8)		
**Fisher’s Alpha**				0.7	< 0.9
Mean (SD)	4.8 (5.0)	4.6 (3.7)	5.1 (3.5)		
**Pielou Evenness**				> 0.9	> 0.9
Mean (SD)	0.4 (0.3)	0.4 (0.2)	0.4 (0.2)		
**Simpson Evenness**				0.5	> 0.9
Mean (SD)	0.1 (0.1)	0.1 (0.1)	0.1 (0.1)		
**Shannon Diversity Index**				> 0.9	> 0.9
Mean (SD)	2.0 (1.6)	1.9 (1.3)	1.9 (1.1)		
**Simpson Diversity**				> 0.9	> 0.9
Mean (SD)	0.5 (0.3)	0.5 (0.3)	0.5 (0.3)		

*Abbreviations*: TX = Texas; PR = Puerto Rico; SD = standard deviation

‡ FDR Adj. P-value indicates a comparison of HPV 16 versus High-risk versus Other

## Data Availability

The raw sequences for the Puerto Rico cohort are available at the European Nucleotide Archive Project (ENA) under the access number ERP136546, and the raw sequences for the Texan cohort will be made available to the public upon publication.
